# Fatty Acid Profiling Identification Method of Emerging Fungal Pathogen *Candidozyma auris* (Formally *Candida auris*)

**DOI:** 10.3390/jof12020130

**Published:** 2026-02-11

**Authors:** Thu Huynh, Flora Bohner, Adiyadolgor Turbat, György Sipos, Attila Gácser, Csaba Vágvölgyi, Tamás Papp, Mónika Varga, András Szekeres

**Affiliations:** 1 Department of Biotechnology and Microbiology, Faculty of Science and Informatics, University of Szeged, Közép Fasor 52, H-6726 Szeged, Hungary; huynh_thu@hcmut.edu.vn (T.H.); flora.bohner@gmail.com (F.B.); adiyadolgor_t@mas.ac.mn (A.T.); gacsera@bio.u-szeged.hu (A.G.); csaba@bio.u-szeged.hu (C.V.); pappt@bio.u-szeged.hu (T.P.); vargam@bio.u-szeged.hu (M.V.); 2 Department of Biotechnology, Faculty of Chemical Engineering, Ho Chi Minh University of Technology (HCMUT), 268 Ly Thuong Kiet Street, District 10, Ho Chi Minh City 72607, Vietnam; 3 Vietnam National University Ho Chi Minh City, Linh Trung Ward, Thu Duc District, Ho Chi Minh City 71351, Vietnam; 4 Laboratory of Microbiology, Institute of General and Experimental Biology, Mongolian Academy of Science, Ulaanbaatar 13330, Mongolia; 5 Functional Genomics and Bioinformatics Group, Faculty of Forestry, University of Sopron, Bajcsy-Zsilinszky Str. 4., H-9400 Sopron, Hungary; sipos.gyorgy@uni-sopron.hu; 6 HUN-REN-SZTE Pathomechanisms of Fungal Infections Research Group, University of Szeged, Közép Fasor 52, H-6726 Szeged, Hungary

**Keywords:** *Candidozyma auris*, chemotaxonomy, fatty acid profiling

## Abstract

The species *Candidozyma auris* (formerly known as *Candida auris*) can be subdivided into four major and two minor clades. It is considered an emerging multidrug-resistant pathogen that causes invasive outbreaks around the world. Therefore, the accurate identification of this species plays an important role in combating invasion and facilitating pathogenic management. In our study an optional identification method was developed considering the possibility of using cellular fatty acids (FAs) as a taxonomic and diagnostic tool. FAs were recorded in the collected *C. auris* strains, and the species characteristic components were determined. Within the isolates examined, the clades were also separated in the statistical analysis. Furthermore, FAs from strains belonging to clade I and II have been divided into two distinct clusters. In testing the performance of the method, all identified samples showed good matches with the established *C. auris* record in the database without misreading. Taken together, cellular fatty acids were investigated as potential discriminatory biomarkers. The results suggest that this approach can distinguish *C. auris* from related species and provides distinctive fatty acid profiles for the investigated *C. auris* clades. The present findings revealed the first report on the application of whole cell FA components as taxonomic features in *C. auris*.

## 1. Introduction

*Candidozyma auris* (formerly known as *Candida auris*) is an emerging multidrug-resistant fungal pathogen first isolated in 2009 from an external ear canal in Japan [1]. Since its discovery, genomic investigations have identified six distinct geographical clades: South Asian (I), East Asian (II), African (III), South American (IV), Iranian (V), and Southeast Asian (VI) [2,3,4]. *C. auris* has rapidly become a global healthcare threat, causing outbreaks of invasive candidiasis, with reported mortality rates ranging from 30% to 60% [5,6,7,8]. The pathogen displays a remarkable capacity for persistence on healthcare surfaces and can be isolated from a wide array of clinical sites, including the bloodstream, respiratory tract, and deep tissues [5,6,9]. A defining and alarming characteristic of *C. auris* is its extensive antifungal resistance profile. It exhibits significantly higher rates of fluconazole resistance compared with most other *Candida* species [5]. Furthermore, the emergence of pan-resistant isolates—resistant to all three major classes of antifungals (azoles, polyenes, and echinocandins)—severely limits therapeutic options [8,10]. To guide clinical management, the US Centers for Disease Control and Prevention (CDC) has established tentative minimum inhibitory concentration (MIC) breakpoints for key antifungal agents [11].

Despite its clinical significance, the management of *C. auris* is hindered by significant diagnostic challenges. Conventional biochemical platforms frequently misidentify *C. auris* as phylogenetically related species such as *C. famata*, *C. guilliermondii*, *C. lusitaniae*, *C. parapsilosis*, *C. intermedia*, *C. catenulata*, *C. haemulonii*, *C. sake*, *C. duobushaemulonii*, *Rhodotorula glutinis*, and *Saccharomyces kluyveri* [6,12,13,14]. While advanced biochemical technologies such as API 20C, Vitek 2, Phoenix, and MicroScan; molecular-based tools including polymerase chain reaction (PCR), loop-mediated isothermal amplification (LAMP), and T2 magnetic resonance (T2MR); as well as mass spectrometric techniques using matrix-assisted laser desorption/ionisation time-of-flight analysis (MALDI-TOF) have improved diagnostic accuracy, their implementation is often limited by high costs and the requirement for specialised laboratory infrastructure [5,15,16,17,18]. Consequently, there is a critical need for accessible, robust, and cost-effective alternative methods for the rapid identification of this pathogen.

The whole cell fatty acid (FA)-based typing system using gas chromatography (GC) could also be a promising alternative for the identification of this species. The Sherlock chromatographic analysis system (CAS) provides a standardised framework for this approach, and FA biomarkers have previously been used for the identification of various *Candida* species [19,20,21]. However, the present study is the first report on an examination of whole cell FA components in *C. auris* across multiple clades. Our objective was to lay the foundations for robust and reliable identification of isolates belonging to this global priority pathogen.

## 2. Materials and Methods

### 2.1. Strains and Cultivation Conditions

The *C. auris* strains used in this study are listed in Table 1. The information followed the ‘*C. auris* Panel’ offered by the CDC & FDA Antibiotic Resistance (AR) Isolate Bank, which contains *C. auris* isolates from all clades [22]. Additionally, MMC-1 and MMC-2 were provided by the Montefiore Medical Center (MMC, Bronx, NY, USA) [23,24]. The *C. albicans* ATCC 14053 was purchased from the American Type Culture Collection (ATCC). Before FA profiling, to ensure metabolic stability and profile standardisation according to the Sherlock MIS protocols, strains were inoculated with the quadrant streaking method on Sabouraud dextrose agar (SDA, BD-Difco, Franklin Lakes, NJ, USA) and incubated at strictly controlled conditions of 28 °C for 30 ± 2 h [25].

### 2.2. The Fatty Acid Methyl Ester (FAME) Analysis

The MIDI Sherlock^®^ Microbial Identification System (MIS, Microbial ID Inc., Newark, NJ, USA) was applied for data acquisition [25]. The composition of whole cell FAs was determined by Sherlock CAS Software ver. 6.4 (Microbial ID Inc., Newark, NJ, USA) operating through the LabSolution ver. 5.97 software (Shimadzu, Kyoto, Japan) on a Nexera GC-2030 gas chromatograph (Shimadzu, Kyoto, Japan) equipped with an AOC-20i Plus autoinjector (Shimadzu, Kyoto, Japan). For the separation of the FAs, the manufacturer provided the SYEAST6 method, which was applied on a HP-Ultra 2, 25 m × 0.2 mm × 0.33 µm film thickness fused silica capillary column (Agilent, Palo Alto, CA, USA) using injector and detector temperatures of 250 °C and 300 °C, respectively. The carrier gas was hydrogen at a flow rate of 0.42 mL/min, while detector gases were nitrogen (make up), oxygen and hydrogen with a follow flow of 30 mL/min, 30 mL/min and 350 mL/min, respectively. The injection volume was 2.0 µL, which was introduced in split mode with a split ratio of 45:1. The oven programme started at 170 °C, which increased to 260 °C with 5 °C/min, and then up to 300 °C with 60 °C/min, holding at this temperature for 2 min. The total programme time of the column oven was 20.67 min. The 1300 C rapid calibration standard mix (Microbial ID Inc., Newark, NJ, USA) was used for retention time calibration and system suitability purposes. *C. albicans* ATCC 14053 and pure hexane were considered as the quality and negative control, respectively. The whole cell FAME profiles were analysed by the library YST28 and YSTCLN (Microbial ID Inc., Newark, NJ, USA). The summed features (SFs) represent groups of two or three fatty acids that could not be separated using the MIDI system. Summed feature 8 (SF8) comprises 18:1 Cis 9 (ω9) and 18:1 (ω8); summed feature 10 (SF10) comprises 18:1 Cis 9 DMA and a sub-peak with ECL 18.218.

### 2.3. Sample Pretreatment

Sample processing was carried out according to the Sherlock^TM^ operating CAS Manual [25]. Briefly, 20–40 mg of cells was harvested and placed in a clean glass tube. Then 1 mL of reagent 1 (45 g NaOH, 150 mL of methanol and 150 mL of distilled water) was added to the sample and heated at 95–100 °C in a water bath (precision water bath NB-301, HandyLAB^®^ System (N-BIOTEK Inc., Gyeonggi-do, Bucheon-si, Republic of Korea). After 5 min, the sample was removed from the water bath, then vortexed and heated for an additional 25 min. The sample was mixed with 2.0 mL of reagent 2 (325 mL of 6.0 N HCl, 275 mL of methanol) and incubated at 80 °C in a water bath for 10 min. After that, 1.25 mL of reagent 3 (200 mL of hexane, 200 mL of methyl tertbutyl ether) was added and the derivatised FAs were extracted for 10 min in a laboratory rotator (Stuart STR4, Cole-Parmer^TM^, Vernon Hills, IL, USA). The organic (upper) phase was recovered and washed with 3.0 mL of reagent 4 (10.8 g NaOH, 900 mL of distilled water) for 5 min on a laboratory rotator. The resulting organic (upper) phase from the tube was transferred to a clean vial for GC analysis.

### 2.4. Statistical Analysis

Each peak from the chromatographic analysis was listed by retention time (Rt), response, equivalent chain lengths (ECLs) and area/height ratio (AR/HT). Sherlock’s peak naming methodology used the composition of the calibration standard to continually monitor the accuracy of system. The CAS automatically calculated a “nominal RT” for each peak based on entries into the peak naming table ECL values for all of peaks in the calibration mix permits. The Sherlock CAS compared the ECL of each peak in the analysis with the expected ECL of the FAs in the peak naming table. Compounds eluted from the column at ECL values below those of known compounds would be assigned an interpolated ECL value. The FA name was printed in the peak name column. Peaks that did not correspond to the ECL values of known FA peaks were left unnamed and were not used in the library search. The ECL value of the peak corresponding to other FAs that could not be resolved reliably from another FA using the chromatographic conditions chosen comprised a portion of an SF. The total percentage of all FAs that were grouped as one feature was printed at the end of the FA composition list [25].

Once a sample had been analysed by Sherlock, its FA composition could be matched with that of known organisms that were stored in libraries. The naming of the unknown was available immediately upon completion of the analysis. The Sherlock library searching lists the most likely matched to the unknown composition and provides a similarity index (SI) for each match. The SI is a numerical value that expresses how closely the FA composition of an unknown compares with the mean FA composition of the strains used to create the library entry listed as its match. The database searched presents the best matches and associated SI. This value was a software-generated calculation of the distance, in multidimensional space, between the profile of the unknown and the mean profile of the closest library entry.

The library generation function of MIS Sherlock Command Centre ver. 6.4 was applied to install a new library of *C. auris*. New entries of *Candida* species were added by a statistical summary of a set of related samples. The MIS Sherlock Command Centre was applied for data analysis. The dendrogram cluster analysis technique, using the Euclidian distance (ED) metric to determine the distance between individual FAs, produced unweighted pair matchings based on FA compositions. The results were displayed graphically to depict the relationship between the organisms. The 2-D plot cluster analysis technique used principal component analysis (PCA) to separate groups of samples in n-dimensional space.

## 3. Results

### 3.1. FAME Profiles of C. auris

The main features of FAs constructed from the MIS analysis of twelve strains (*n* = 10) were revealed and the profiles varied among the strains (Table 2). While the total number of isolates analysed was relatively small, all strains were obtained from the CDC & FDA Antibiotic Resistance (AR) Isolate Bank (*C. auris* Panel), which was curated to represent the major genetic diversity and geographic clades (I–IV) of *C. auris* circulating worldwide. Despite the limited number of isolates per clade, the inclusion of these reference strains captured core fatty acid profiles of the major lineages, and technical replication demonstrated high stability and reproducibility of the fatty acid profiles. The FA 16:0, 18:2 Cis 9,12/18:0a and SF8 FAs were predominated within the strains. The visual representation of the fatty acid profiles is provided in the Supplementary Material (Figures S1–S12).

The content of 16:1 Cis 9 (ω7) among the examined strains ranged from 2.84 to 6.68 with the lowest and highest amounts at B11244 (Clade IV) and MMC-2 (Clade II) isolates, respectively. The 16:0 content of strain B11220 (clade II) was lower (13.54%) than others (15.82–20.79%). The strain MMC-2 contained 10.51% of 17:1 Cis 9 (ω8), which was remarkably less in other isolates, ranging only within 1.94–4.80%. Nevertheless, the strains B11220 (clade II) and B11244, B11245 (clade IV) shared a similar ratio, with the lowest 18:2 CIS 9,12/18:0a (27.25–28.41%) and the highest SF8 (40.87–43.08%). Compared with strains of clade II and IV, strains B11221 and B11222 (clade III) contained higher 18:2 CIS 9,12/18:0a (29.56–30.08%) but lower SF8 (37.48–38.04%) except for MMC-2 (clade II), showing moderately high 18:2 CIS 9,12/18:0a and the lowest level of SF8 (Table 1). The strains B11109 and B8441 of clade I shared a similar ratio of 18:2 CIS 9,12/18:0a (27.86–28.57%) but lower SF8 (35.67–35.80%). On the contrary, the content of 18:2 CIS 9,12/18:0a (35.96–39.39%) in the other strains of clade I such as B11098, B11203, AR0390, and MMC-1 shared a higher content of SF8 (30.64–33.1%).

High intra-sample reproducibility was observed across the 10 technical replicates for each strain, as reflected by the low SD values reported in Table 2. For the majority of fatty acids, SD values were below 0.5%, indicating a high level of technical stability of the method. Accordingly, the quantitative differences observed between strains and clades, while sometimes small in magnitude, were larger than the technical variability captured under the applied experimental conditions.

### 3.2. Identification of FAME Subgroups of C. auris Species

**Figure 1:**
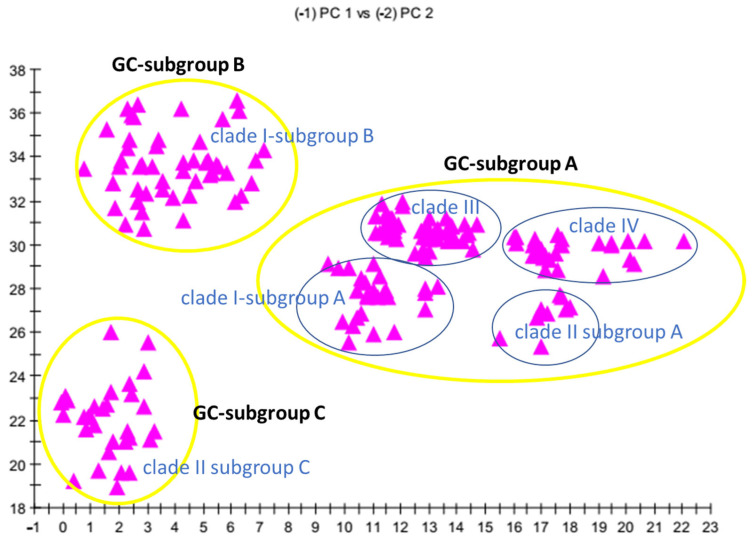
Principal component analysis (PCA) of whole cell fatty acid methyl ester (FAME) profiles of the analysed *C. auris* isolates. The first two principal components (PC1 and PC2) were generated using the MIS Sherlock Command Centre software (version 6.4). Outlines indicate clade or GC subgroup assignment. PCA is presented as an exploratory visualisation of similarity patterns rather than as a classification tool.

**Figure 2:**
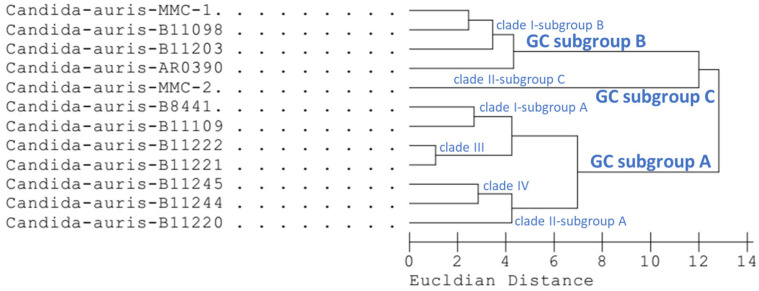
The relationship of FA profiles in *C. auris* clades and GC subgroups generated by MIS Sherlock Command Centre software (version 6.4). The Euclidean distance is shown on the *x* axis at the bottom of the figure.

Cluster and PC analysis of the resulting FAME profiles (Table 2 and Table 3) drew a distinction dividing 12 strains of *C. auris* into three gas chromatographic (GC) subgroups (Figure 1 and Figure 2). The GC subgroup A included B11220, B11109, B11221, B11222, B11244, B11245 and B844 strains. The GC subgroup B contained B11098, B11203, AR0390 and MMC-1 isolates, while the GC subgroup C involved only the MMC-2 strain. This subgroup is represented by a single isolate and therefore reflects strain-specific variation rather than a taxonomically defined subgroup.

The fatty acid profiles were consistently characteristic and showed exploratory differences among the three groups. PCA was applied as an exploratory visualisation tool to reduce data dimensionality and to visualise the main trends and group separations in two- or three-dimensional spaces, complementing the quantitative similarity analysis provided by the MIS software (version 6.4). The 2D plot built from PC 1 and PC 2 (Figure 1) showed a separation of the FA patterns in an n-dimensional space, where the ED2 of subgroups A, B and C were ~100, ~72, and ~13.5, respectively. These ED values indicate a substantial quantitative separation between the groups in the multidimensional space, exceeding the separation typically observed among closely related biochemical phenotypes.

The FA 17:1 Cis 9 (ω8), 17:0, 18:2 Cis 9,12/18:0a and SF8 were the biomarkers between three GC subgroups of *C. auris* (Table 3). The lower content of 17:1 Cis 9 (ω8) and 17:0 in subgroup A (2.84 and 1.79%) and subgroup B (3.12 and 1.01%) can be distinguished from subgroup C (10.51 and 3.81%). Additionally, subgroup A contains a lower proportion of 18:2 Cis 9,12/18:0a (28.47%) and a higher proportion of SF8 (38.85%) than two other subgroups, whereas subgroups B and C contain a higher proportion of 18:2 Cis 9,12/18:0a (37.08 and 30.20%) and a lower proportion of SF8 (31.39 and 26.43%) than subgroup A. The different amounts between 18:2 Cis 9,12/18:0a and SF8 have also drawn discrimination between groups. The repeated FA profile analysis of the available isolates provided good matches at the subgroup level (SI > 0.7) using the FA patterns created from the results of dendrogram analysis by MIS software. The visual representation of the subgroup profiles is provided in the Supplementary Material (Figures S13–S15).

### 3.3. FA Compositions in C. auris Clades

Our results (Table 4, Figure 1) revealed that the represented *C. auris* clades also provided distinct FA compositions.

As revealed, the *C. auris* clades shared similar FA components qualitatively, but showed differences in their quantities. The differences in proportions of FAs in Table 4 served as differentiating features among the examined *C. auris* isolates also at the clade level.

The MIS software also contains a dendrogram program that compares the similarity of the fatty acid profile of the yeasts identified by the system and establishes the degree of relatedness of microorganisms at the subgroup, clade, species or genus level. To determine the relationship, a dendrogram was created that exhibits the FA profiles of the examined *C. auris* strains (Figure 2). In some cases, strains within certain clades had shared similar FA profiles such as clade, clade III that includes strains B11221 and B11222, and clade IV that includes strains B11244 and B11245 grouping into distinct clusters on the dendrogram. On the other hand, the isolates belonging to clade I (B11109, B8441, B11098, B11203, AR0390 and MMC-1) and clade II (B11220 and MMC-2) were clustered into the separate branches of the dendrogram forming the clade I subgroup A and B as well as the clade II subgroup A and C. Interestingly, the FA composition of the MMC-2 strain (clade II) was significantly divergent to others, forming a completely separated cluster named subgroup C, including only the MMC-2 isolate. Based on the repeated FA profile analysis of the available isolates, the clade level classifications showed good matches (SI > 0.7) using the FA patterns created from the results of dendrogram analysis by MIS software. The visual representation of the clade profiles is provided in the Supplementary Material (Figures S16–S21).

### 3.4. Application of FAME Profiles as a Taxonomic Biomarker in C. auris

Analysis of whole cell FAs using MIS for identification in yeast has been used as an alternative for taxonomic purposes [19,20,21]. This study aimed to provide a comprehensive illustration of whole cell FA profiles of *C. auris* to complete the knowledge about their taxonomical relationships and to highlight the potential as a supplementary method to identify these human pathogenic yeasts. In this study, the library was carefully generated, including FA components of clades and GC subgroups based on FA components of 12 strains of *C. auris*. The number and frequency of FAs using library training files were *n* = 10 for each strain. In addition, the standard libraries YST28 and YSTCLN containing other yeast and *Candida* species constructed by MIDI were also included into the evaluation [22].

**Figure 3:**
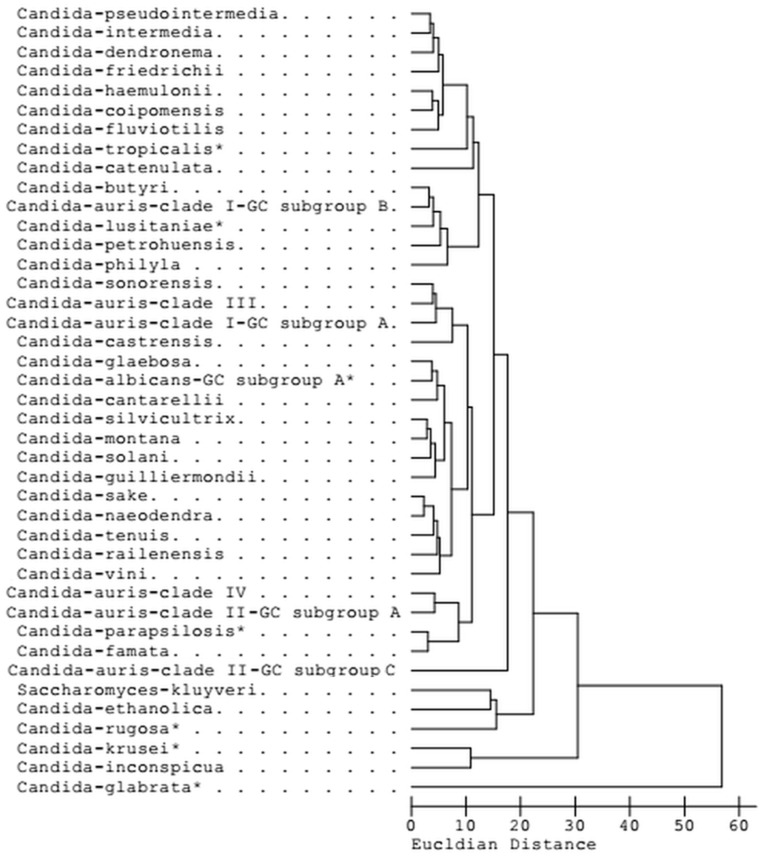
The relationship of fatty acid (FA) profiles among yeast species included in the YST28 and YSTCLN libraries was analysed using the MIS Sherlock Command Centre software (version 6.4). Clinical species other than *C. auris* are indicated by an asterisk, and Euclidean distance values are displayed on the *x*-axis at the bottom of the figure.

For testing the performance of the new libraries, the available strains were chromatographically analysed, and the SI values were calculated based on the obtained FA pattern, indicating the goodness of the taxonomical fits. In our case, all identified samples exhibited good matches to *C. auris* (SI > 0.7) without misreading. Additionally, well-SI separations (>0.1) were observed between our *C. auris* isolates and other generated species in libraries YST28 and YSTCLN, confirming that the method is reliable as exploratory observations (Figure 3). Furthermore, FA profiles of clades were also distinguishable within the examined *C. auris* isolates (Figure 2). Within the clades represented by the examined strains, significant library matches (SI > 0.7) were found; the separation to each other was also significantly high (>0.1). During internal validation using the generated library entries, no misassignments were observed within the examined dataset. Across repeated confirmation measurements (*n* = 120 analyses), no isolate was incorrectly assigned to a different clade, and all valid matches yielded similarity index (SI) values above 0.7. These results indicate high internal consistency under the applied experimental conditions. Within clades, FA profiles revealed minimal library differences (SI > 0.7) and narrow-SI-separation (<0.1), confirming the significantly high similarity of clade members’ FA profiles.

The dendrogram constructed from diverse FAME profiles of *C. auris* and related species illustrates phenotypic relationships based on Euclidean distance (ED). The UPGMA-based chemotaxonomic clustering reveals pronounced separation between major groups, as reflected by the relatively large ED intervals observed between clusters (Figure 3). In particular, the branching of *C. auris* clades from related *Candida* species occurs at higher ED values (above 20), indicating marked differences in fatty acid composition under the applied experimental conditions. These profiles were obtained from the Sherlock library YST28 that had been built from more than 20 various strains within each species. The samples were collected from around the world to avoid potential geographic bias and carefully analysed with many replications to make highly representative library entries [22]. The FA profiles of clade I—GC subgroup A strains formed a tight cluster close to *C. sonorensis* together with clade III isolates; the FA profiles of clade I—GC subgroup B also formed another cluster close to *C. butyri*, *C. lusitaniae*, *C. petrohuensis* and *C. philyla*. Furthermore, the FA profiles of the clade IV clustered to the clade II—GC subgroup A, while clade II—GC subgroup C formed a significantly separated cluster branched with a high ED value from the neighbouring node. Efforts to investigate the application of FAME profiles as taxonomic markers showed potential discriminatory power not only between *C. auris* and related species but also at the clade level, at least among the examined four *C. auris* clades, as exploratory observations (Figure 3). Prior to fatty acid profiling, strains were inoculated onto Sabouraud dextrose agar using the quadrant streaking method and incubated at 28 °C for 30 ± 2 h.

## 4. Discussion

Currently, *C. auris* has diverged into four major and two minor clades [2,3,4]. The clades are specific to each geographic region displaying distinct biological and drug resistance properties: clade I (South Asian), clade II (East Asian), clade III (South African), clade IV (South American), clade V (Iran) and clade VI (Singapore) [2,3,4]. An average pairwise nucleotide identity of 98.7% between genomic assemblies of clades was observed [8]. In addition, the high genetic diversity between clades was discriminated by 10,000 single-nucleotide polymorphisms (SNP) [23].

Clade-typing largely based on time-consuming whole genome sequencing and multilocus sequence typing [23,24,25], as well as the clade-specific identification using short tandem repeats (STRs), involves a sequencing step [26]. Allele-specific PCR (AS-PCR) based on conserved mutations in the internal transcribed spacer sequences reported recently [27] relies on multiple reactions to differentiate the clades properly, while a colony PCR-based clade-identification system (ClaID) is also able to distinguish rapidly between the four major clades of *C. auris* [28]. Given that the classification of *C. auris* at clade level by genetic platforms requires in-depth genetic knowledge and significant bioinformatics expertise, the MALDI-TOF MS system can rapidly and accurately identify *C. auris* and related species, making it a key method for the accurate diagnosis of infectious agents of the genus *Candidozyma* in clinical practice [29]. Based on the literature reports, the application of the MALDI-TOF MS technique is able to accurately identify *C. auris* and members of the *C. haemuli* species complex [25,30] and successfully differentiates the *C. auris*, *C. haemuli*, *C. haemuli* var. *vulnera*, and *C. duobushaemuli* [15]. Furthermore, a MALDI-TOF database was also built by Zhu et al. for *C. auris* and all of its phylogenetically related species of the *C. haemuli* species were successfully determined [29]. PCR techniques, particularly those aimed at targeting ribosomal DNA and internal transcribed spacer (ITS) regions, have shown rapid and reliable identification capabilities for *C. auris* [6,31,32]. Real-time PCR has been validated to recognise all major clades with commendable sensitivity and specificity, proving particularly useful in outbreak scenarios [32,33]. WGS provides the most detailed characterisation of *C. auris* isolates, allowing researchers not only to confirm species identification but also to understand genetic diversity and resistance mechanisms [14,25]. While whole-genome sequencing of *Candidozyma auris* is often reserved for outbreak investigations, findings from this retrospective genomic analysis demonstrate that WGS can also provide actionable information to describe the evolution and transmission of *C. auris* over a defined interval and to assess the establishment of endemicity [34]. However, the cost and infrastructure required to implement WGS on a routine basis may limit its practicality compared with more accessible methods such as MALDI-TOF MS or PCR for immediate clinical diagnostics [35]. While LAMP is less frequently discussed in the literature compared with MALDI-TOF MS and PCR for *C. auris* identification, its potential lies in providing rapid diagnostics. LAMP’s inherent advantages include simplicity and sensitivity under isothermal conditions [35]. The LAMP approach has been shown to reliably identify all tested *C. auris* strains directly from clinical specimens within a short time, without technical complications related to instrument use. By eliminating the need for cultivation and DNA extraction, this method enables earlier diagnosis and supports timely clinical decision making [36]. This method, termed LAMPAuris, was later validated for its ability to identify *C. auris* isolates representing five distinct clades, even when directly applied to clinical specimens [37]. In summary, while MALDI-TOF MS and PCR stand out as the most reliable and practical methods for identifying *C. auris* and its clades, emerging technologies like LAMP and WGS present promising potential that may need further exploration and validation. Continuous advancements in diagnostics and integration of technologies will be essential in the ongoing battle against this formidable pathogen.

The MIS system analyses by identifying and quantifying long-chain FAs of 9 to 20 carbon atoms of samples compared with those of well-characterised strains in the database library for identification [22]. In general, the three subgroups of FAs in *C. auris* have some differences compared with other *Candida* species in previous reports (Table 5). *C. auris* contains FA 17:0, which is lacking in other reported profiles (Table 5). Furthermore, the proportion of 18:2 Cis 9,12/18:0a in *C. auris* is higher, while those of 18:0 and SF8 are lower than those of other species. To make a comparison between *C. auris* and relative species, FA profiles of species in the commercial library YST28 created by MIDI [22] were referenced.

Consequently, the FA compositions detected in *C. auris* were qualitatively similar but quantitatively different from other *Candida* species, as revealed. FA profiles of GC subgroup A and neighbouring *C. sonorensis* share a cluster. However, *C. auris* GC subgroup A contains a lower proportion of 16:0 and 18:2 Cis 9,12/18:0a and a higher proportion of 17:0 and SF8 than relative species. Compared with closely related *C. butyri*, *C. lusitaniae*, *C. petrohuensis*, *C. philyla* and *C. castrensis*, GC subgroup B contains a higher proportion of 17:1 Cis 9 (ω8) and a different proportion of 16:1 Cis 9 (ω7), 16:0, 18:2 Cis 9,12/18:0a, 18:0 and SF8. Furthermore, frequent misidentification cases of *C. auris* such as *C. famata*, *C. guilliermondii*, *C. lusitaniae*, *C. parapsilosis*, *C. intermedia*, *C. catenulata*, *C. haemulonii*, *C. sake*, and *S. kluyveri* [6,12] phylogenetically related to *C. auris* such as *C. catenulate*, *C. ethanolica*, *C. glabrata*, *C. guilliermondii*, *C. haemulonii*, *C. inconspicua*, *C. intermedia*, *C. krusei*, *C. lusitaniae*, *C. parapsilosis*, *C. rugosa*, and *C. tropicalis* [1,8,23] are also distinguished into distinct clusters by FAMEs (Figure 3). The present investigation demonstrates the potential application of FA compositions as taxonomic biomarkers of *C. auris* at the species level.

The *C. auris* species belongs altogether in six clades at present [4], four of them represented in our present study. As observed in Table 4, clade I—GC subgroup B is distantly related to the clades of GC subgroup A by a higher proportion of 18:2 Cis 9,12/18:0a and a lower proportion of SF8 (including 18:1 Cis 9 (ω9) and 18:1 (ω8)). Similarly, based on the WGS analysis, strain B8441 (clade I—GC subgroup A) and strain B11098 (clade I—GC subgroup B) are divergent to two subbranches of clade I [23].

The FA composition of strain MMC-2 (clade II) is significantly divergent to others that form a distinct cluster named subgroup C. Subgroup C contains a significantly higher content of 17:1 Cis 9 (ω8) and a lower content of SF8. Previously, discriminated traits between strains MMC-2 and MMC-1 were already investigated such as resistance to fluconazole and lipid biosynthesis [38,39] and their taxonomical status were recently confirmed via the ClaID system [40]. However, since the ClaID system did not show a taxonomical distance between two clade II isolates (AR0381 and MMC-2), our analysis divided it into two highly separated subgroups.

While the present study demonstrates the potential of FAME profiling as a discriminatory approach, we acknowledge the limitation imposed by the relatively small sample size. Although the number of analysed isolates was limited (*n* = 12), the strains were selected from the CDC & FDA Antibiotic Resistance Isolate Bank to represent the core genetic diversity of the major global *C. auris* clades. Accordingly, the resulting profiles are considered representative of characteristic fatty acid signatures within these lineages.

With respect to the distinct clustering of isolate MMC-2 (clade II) into a separate subgroup, caution is warranted when interpreting this pattern as a general taxonomic feature. However, previous studies have reported unique lipid biosynthesis-related traits and resistance-associated characteristics for MMC-2 compared with other clade II isolates. In this context, the divergence observed in our FAME analysis is consistent with documented biochemical differences and is therefore interpreted as a strain-specific phenotypic variation rather than random analytical noise.

Furthermore, the low standard deviation values observed across technical replicates (Table 2) indicate that the detected fatty acid variations, while quantitatively modest, are stable and reproducible under the applied experimental conditions. Taken together, these findings support the interpretation of the present study as a proof-of-concept, while emphasising that validation using larger and more diverse clinical isolate collections will be required to strengthen statistical power and broader applicability.

While FAME profiling may offer certain advantages in clinical scenarios, it also has practical limitations, including the need for specialised equipment and chemical derivatisation. In our examinations, an identification method based on FAME profiles was successfully developed and the previously described clades of the examined isolates were also distinguished. The literature indicates that molecular methods already enable clade-level identification with superior precision; nevertheless, continued efforts to explore and develop complementary or improved approaches remain warranted. In this context, the MIS, as a cost-effective, sensitive, and reliable system, has been considered a potentially applicable method for the identification of *C. auris*, including at the clade level.

## 5. Conclusions

The present study developed a novel biomarker-based identification method using whole cell FAME together with Sherlock CAS for global invasive *C. auris*, optionally applicable other than the present phenotypic assays, overcoming the shortcomings of some existing ones. As a result, cellular FAs were investigated as a sufficiently discriminatory biomarker that distinguishes not only between *C. auris* and related species but also showed distinctive trends within the *C. auris* clades examined in our study as exploratory observations. To our knowledge, the present findings show the first report on whole cell FA components in *C. auris* and their application as taxonomic features at the species and clade level. Hopefully, our presented results can contribute to successfully combatting this rapidly emerging human pathogenic yeast.

## Figures and Tables

**Table 1:** List of *C. auris* strains used in this study.

Clade	Clade Name	AR Bank/CDC	Strain	Isolation Site	Source
I	South Asia	0382	B11109	burn wound	CDC
		0387	B8441	blood	CDC
		0388	B11098	blood	CDC
		0389	B11203	bronchoalveolar lavage	CDC
		0390	AR0390	wound	CDC
		nr	MMC-1	blood	MMC
II	East Asia	0381	B11220	auditory canal	CDC
		nr	MMC-2	nd	MMC
III	Africa	0383	B11221	blood	CDC
		0384	B11222	blood	CDC
IV	South America	0385	B11244	blood	CDC
		0386	B11245	blood	CDC

nr: not registered in the Ar Bank/CDC, data available, nd: isolation side is unknown.

**Table 2:** Main cellular FA compositions among *C. auris* strains (mean (%) ± SD).

Strain Number	16:1 Cis 9 (ω7)	16:0	17:1 Cis 9 (ω8)	17:0	18:2 Cis 9,12/18:0a	18:0	SF8 *	SF10 ^#^
B11109	5.45 ± 0.64	19.20 ± 0.32	2.88 ± 0.34	1.55 ± 0.27	28.57 ± 0.68	3.00 ± 0.39	35.80 ± 0.79	1.29 ± 0.14
B8441	5.21 ± 0.58	20.79 ± 0.28	3.79 ± 0.72	2.56 ± 0.53	27.86 ± 0.68	3.32 ± 0.31	35.67 ± 0.96	-
B11098	5.49 ± 0.78	20.39 ± 0.40	2.41 ± 0.74	0.53 ± 0.52	35.96 ± 0.61	3.51 ± 0.51	30.94 ± 1.67	-
B11203	4.70 ± 0.36	18.95 ± 0.52	3.21 ± 0.73	-	39.39 ± 1.04	2.56 ± 0.36	30.64 ± 1.17	-
AR0390	3.82 ± 0.72	17.32 ± 0.86	3.28 ± 0.75	2.00 ± 0.44	36.04 ± 0.92	4.03 ± 0.42	33.17 ± 1.24	-
MMC-1	4.69 ± 0.30	18.77 ± 0.60	3.57 ± 1.11	1.17 ± 1.06	36.95 ± 0.52	3.35 ± 029	30.83 ± 1.27	-
B11220	3.10 ± 0.23	13.54 ± 0.44	4.80 ± 0.40	2.42 ± 0.10	27.54 ± 0.63	4.53 ± 0.35	41.04 ± 0.45	0.69 ± 0.10
MMC-2	6.68 ± 0.55	18.96 ± 0.28	10.51 ± 0.56	3.81 ± 0.27	30.20 ± 0.62	3.20 ± 0.31	26.43 ± 0.51	-
B11221	3.64 ± 0.13	18.15 ± 0.27	2.00 ± 0.25	1.44 ± 0.19	29.56 ± 0.29	4.07 ± 0.30	37.48 ± 0.50	1.41 ± 0.25
B11222	3.70 ± 0.05	17.86 ± 0.29	1.94 ± 0.21	1.46 ± 0.13	30.08 ± 0.55	3.52 ± 0.15	38.04 ± 0.38	1.47 ± 0.07
B11244	2.84 ± 0.21	15.82 ± 0.32	2.17 ± 0.34	1.62 ± 0.22	27.25 ± 0.56	4.55 ± 0.24	43.08 ± 1.07	0.76 ± 0.07
B11245	3.50 ± 0.23	16.77 ± 0.34	2.29 ± 0.28	1.47 ± 0.23	28.41 ± 0.39	3.92 ± 0.21	40.87 ± 0.73	0.81 ± 0.09

* Summed feature (SF8) including the peaks of 18:1 Cis 9 (ω9) and 18:1 (ω8), ^#^ summed feature (SF10) including the peaks of 18:1 Cis 9 DMA and a sub-peak with ECL 18.218.

**Table 3:** Cellular FA compositions in three GC subgroups of *C. auris* (mean (%) ± SD).

Subgroups	16:1 Cis 9 (ω7)	16:0	17:1 Cis 9 (ω8)	17:0	18:2 Cis 9,12/18:0a	18:0	SF8 *
GC subgroup A	3.92 ± 1.00	17.45 ± 2.22	2.84 ± 1.08	1.79 ± 0.52	28.47 ± 1.11	3.84 ± 0.62	38.85 ± 2.75
GC subgroup B	4.68 ± 0.82	18.85 ± 1.25	3.12 ± 0.92	1.01 ± 0.96	37.08 ± 1.60	3.36 ± 0.66	31.39 ± 1.67
GC subgroup C	6.68 ± 0.55	18.96 ± 0.28	10.51 ± 0.56	3.81 ± 0.27	30.20 ± 0.62	3.20 ± 0.31	26.43 ± 0.51

* Summed feature (SF8) including the peaks of 18:1 Cis 9 (ω9) and 18:1 (ω8).

**Table 4:** Main cellular FA compositions among *C. auris* clades (mean (%) ± SD).

Clades	GC Subgroup	16:1 Cis 9 (ω7)	16:0	17:1 Cis 9 (ω8)	17:0	18:2 Cis 9,12/18:0a	18:0	SF8 *	SF10 ^#^
Clade I	A	5.33 ± 0.60	19.99 ± 0.87	3.33 ± 0.72	2.06 ± 0.66	28.22 ± 0.76	3.16 ± 0.38	35.73 ± 0.86	0.70 ± 0.66
	B	4.68 ± 0.82	18.85 ± 1.25	3.12 ± 0.92	1.01 ± 0.96	37.08 ± 1.60	3.36 ± 0.66	31.39 ± 1.67	-
Clade II	A	3.10 ± 0.23	13.54 ± 0.44	4.80 ± 0.40	2.42 ± 0.10	27.54 ± 0.63	4.53 ± 0.35	41.04 ± 0.45	0.69 ± 0.10
	C	6.68 ± 0.55	18.96 ± 0.28	10.51 ± 0.56	3.81 ± 0.27	30.20 ± 0.62	3.20 ± 0.31	26.43 ± 0.51	-
Clade III	A	3.67 ± 0.10	18.01 ± 0.31	1.97 ± 0.23	1.45 ± 0.16	29.82 ± 0.50	3.79 ± 0.36	37.76 ± 0.52	1.44 ± 0.18
Clade IV	A	3.17 ± 0.40	16.29 ± 0.58	2.23 ± 0.31	1.55 ± 0.23	27.83 ± 0.76	4.24 ± 0.39	41.97 ± 1.44	0.79 ± 0.08

* Summed feature (SF8) including the peaks of 18:1 Cis 9 (ω9) and 18:1 (ω8), ^#^ summed feature (SF10) including the peaks of 18:1 Cis 9 DMA and a sub-peak with ECL 18.218.

**Table 5:** Cellular FA components in *C. auris* and other species.

	12:0	14:0	15:0	16:1 Cis 9 (ω7)	16:0	C16:0 2OH	17:1 Cis 9 (ω8)	17:0	18:2 Cis 9,12/18:0a	18:0	SF8 *
*C. auris* GC subgroup A	-	0.32	-	3.92	17.45	-	2.84	1.79	28.47	3.84	38.85
*C. auris* GC subgroup B	-	-	-	4.68	18.85	-	3.12	1.01	37.08	3.36	31.39
*C. auris* GC subgroup C	-	-	-	6.68	18.96	-	10.51	3.81	30.20	3.20	26.43
*C. albicans* [21]	-	0.91	-	8.41	14.96	-	1.66	-	27.85	4.20	41.43
*C. dubliniensis* [21]	-	0.50	-	8.91	15.01	-	2.22	-	18.65	5.70	47.31
*C. digboiensis* [19]	0.48	1.48	0.48	5.37	25.23	0.83	0.72	-	19.50	5.22	40.68

* Summed feature including the peaks of 18:1 Cis 9 (ω9) and 18:1 (ω8).

## Data Availability

Dataset available on request from the authors.

## Supplementary Materials

The following supporting information can be downloaded at: https://www.mdpi.com/article/10.3390/jof12020130/s1, Figures S1–S12: Cellular FA compositions of examined *C. auris* strains; Figures S13–S15: Cellular FA compositions of investigated *C. auris* GC subgroups; Figures S16–S21: Cellular FA compositions of represented *C. auris* clades.
