# Supplementary material for: Fatty Acid Profiling Identification Method of Emerging Fungal Pathogen Candidozyma auris (Formally Candida auris)
Source: J Fungi (Basel). 2026 Feb 11;12(2):130. doi: 10.3390/jof12020130 (PMC12941525; doi:10.3390/jof12020130)
Supplement: Supplementary file 1 [file jof-12-00130-s001.zip › jof-4048976-supplementary.pdf]

Fatty acid profiling identification method of emerging fungal pathogen *Candidozyma auris* (formally *Candida auris*)

Thu Huynh [1,2,3], Flóra Bohner [1], Adiyadolgor Turbat [1,4], György Sipos [5], Attila Gácser [1], Csaba Vágvölgyi [1], Tamás Papp [1,6], Mónika Varga [1] and András Szekeres [1,*]

1 Department of Biotechnology and Microbiology, Faculty of Science and Informatics, University of Szeged, Szeged, Közép fasor 52., H-6726 Szeged, Hungary; huynh_thu@hcmut.edu.vn (T.H.); flora.bohner@gmail.com (F.B); adiyadolgor_t@mas.ac.mn (A.T.); gacsera@bio.u-szeged.hu (A.G.); csaba@bio.u-szeged.hu (C.V.); pappt@bio.u-szeged.hu (T.P.); vargam@bio.u-szeged.hu (M.V.); szandras@bio.u-szeged.hu (A.Sz.)
2 Department of Biotechnology, Faculty of Chemical Engineering, Ho Chi Minh University of Technology (HCMUT), 268 Ly Thuong Kiet Street, District 10, 72607, Ho Chi Minh City, Vietnam; huynh_thu@hcmut.edu.vn (T.H.)
3 Vietnam National University Ho Chi Minh City, Linh Trung Ward, Thu Duc District, 71351, Ho Chi Minh City, Vietnam; huynh_thu@hcmut.edu.vn (T.H.)
4 Laboratory of Microbiology, Institute of General and Experimental Biology, Mongolian Academy of Science, Ulaanbaatar, Mongolia-13330; adiyadolgor_t@mas.ac.mn (A.T.)
5 Functional Genomics and Bioinformatics Group, Faculty of Forestry, University of Sopron, Bajcsy-Zsilinszky str. 4., H-9400 Sopron, Hungary; sipos.gyorgy@uni-sopron.hu (G.S.)
6 HUN-REN-SZTE Pathomechanisms of Fungal Infections Research Group, University of Szeged, Közép fasor 52, H-6726, Szeged, Hungary; pappt71@gmail.com (T.P.)
* Correspondence: szandras@bio.u-szeged.hu (A.Sz.).

Supplementary materials

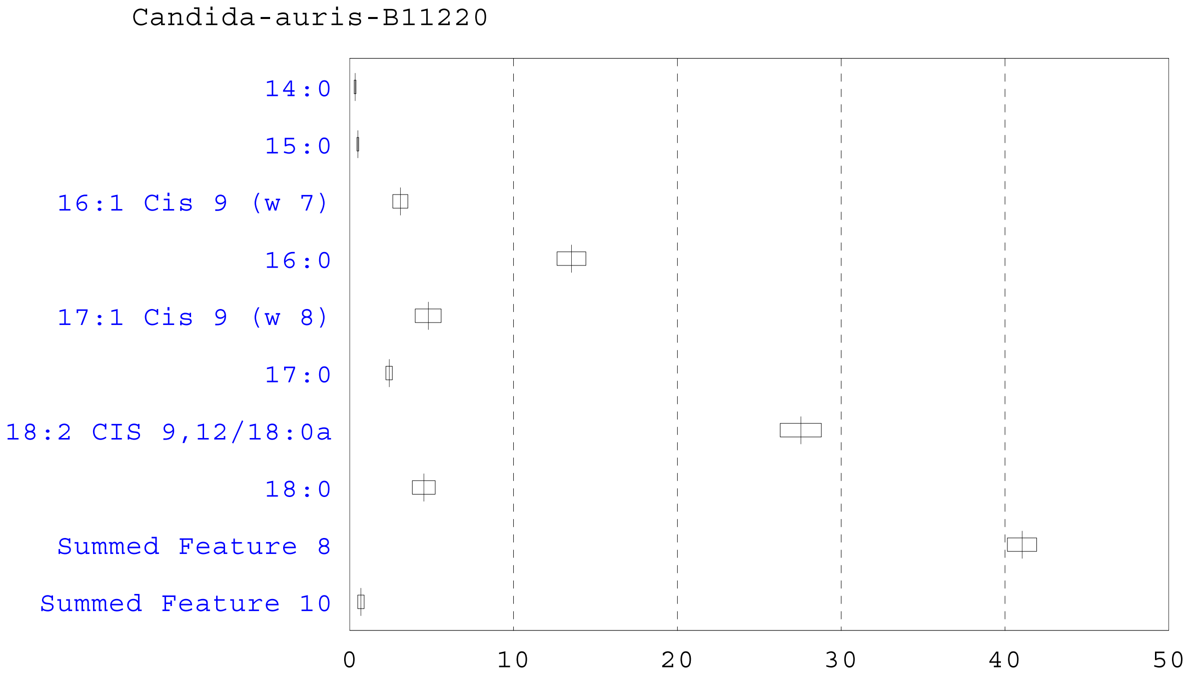

Figure S1. Cellular FA compositions of *C. auris* strain B11220 (Mean (%) ± SD)

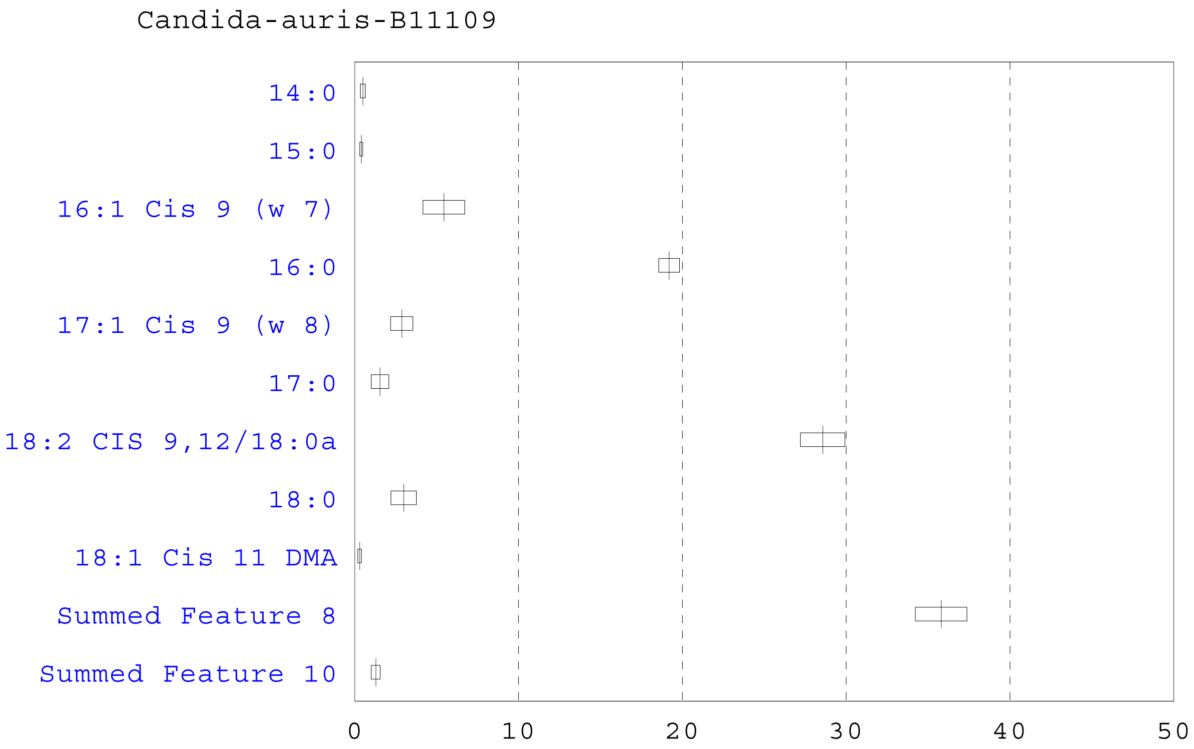

Figure S2. Cellular FA compositions of *C. auris* strain B11109 (Mean (%) ± SD)

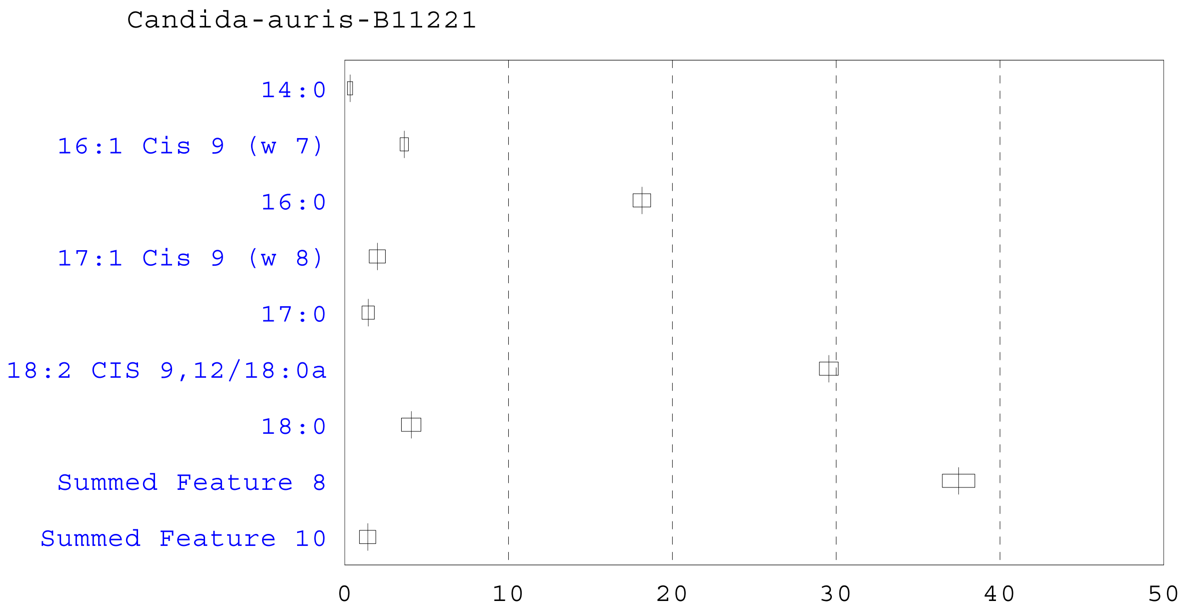

Figure S3. Cellular FA compositions of *C. auris* strain B11221 (Mean (%) ± SD)

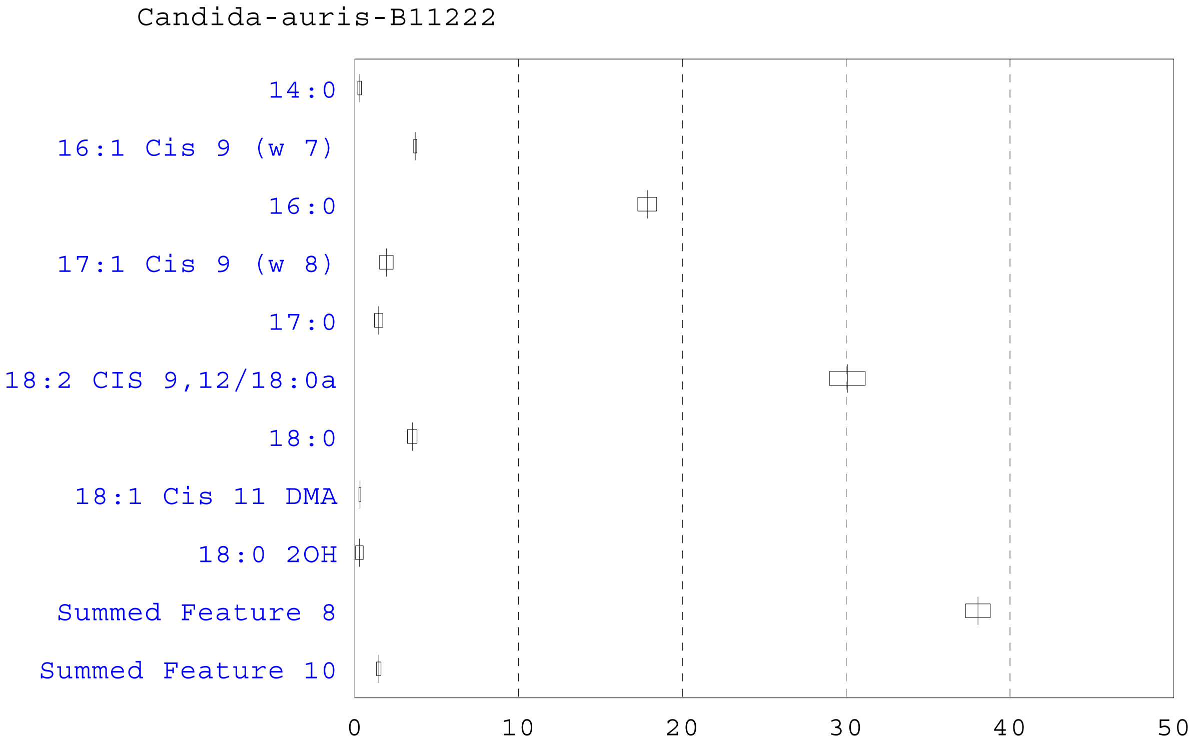

Figure S4. Cellular FA compositions of *C. auris* strain B11222 (Mean (%) ± SD)

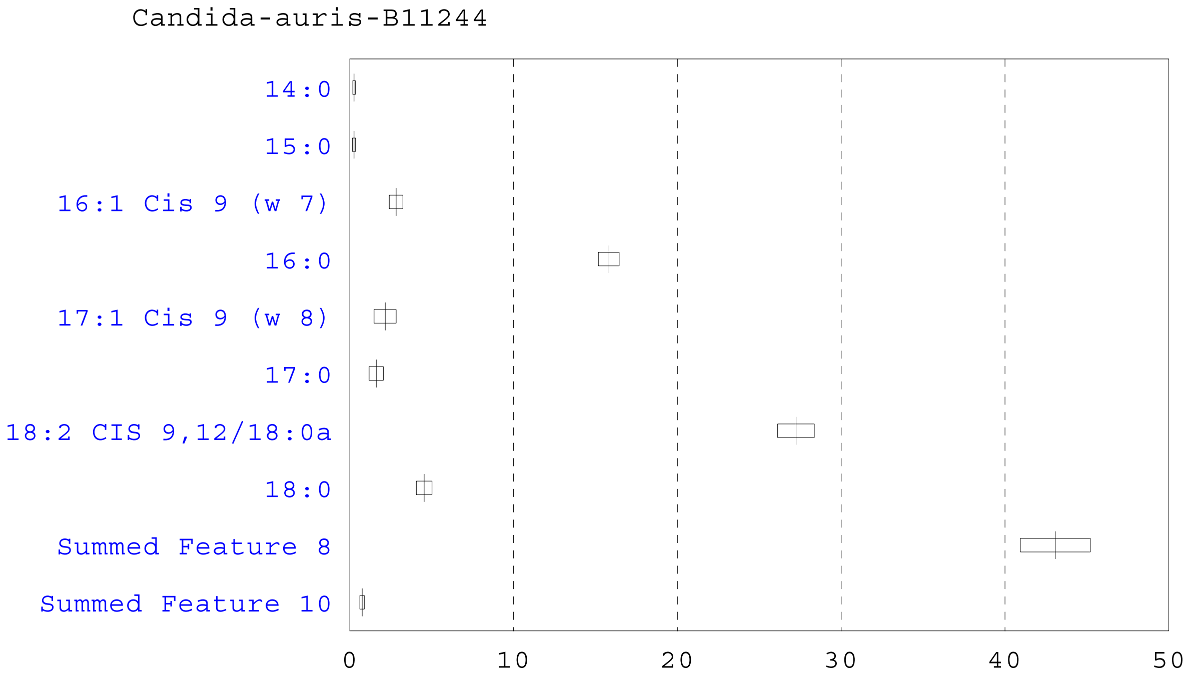

Figure S5. Cellular FA compositions of *C. auris* strain B11244 (Mean (%) ± SD)

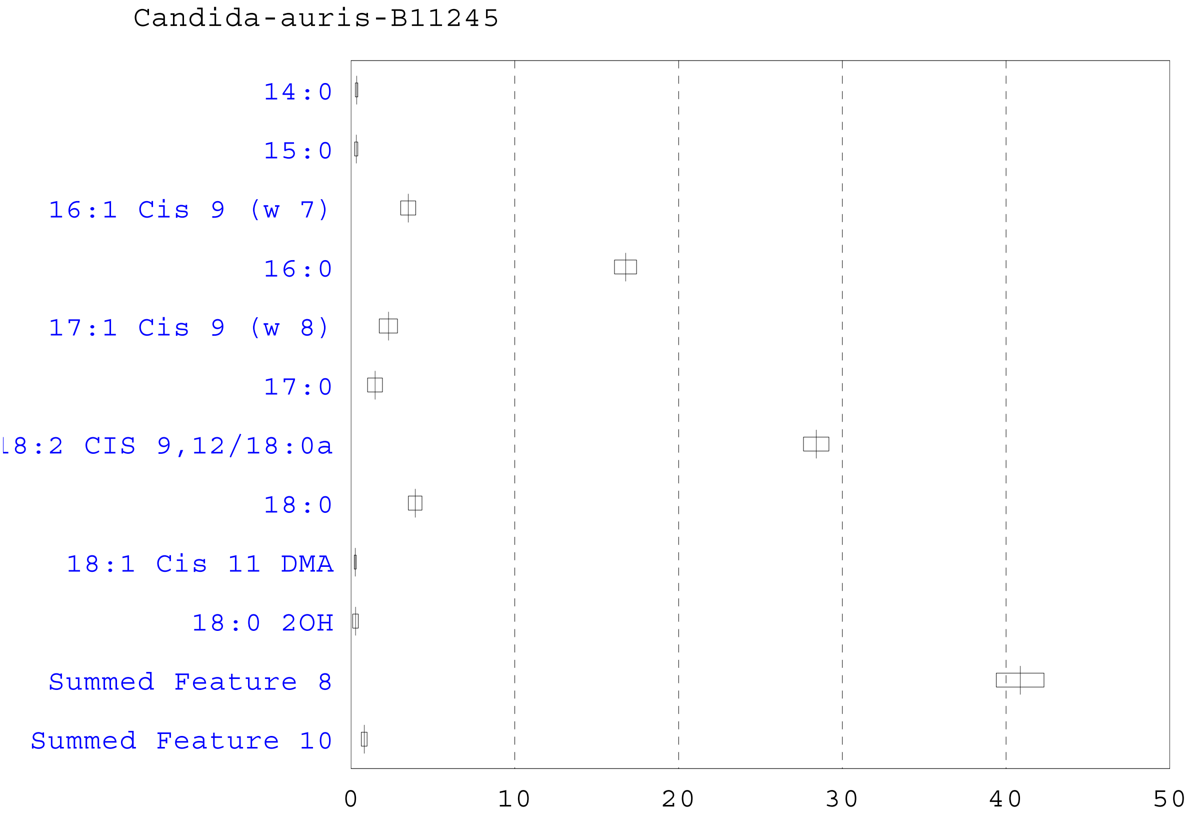

Figure S6. Cellular FA compositions of *C. auris* strain B11245 (Mean (%) ± SD)

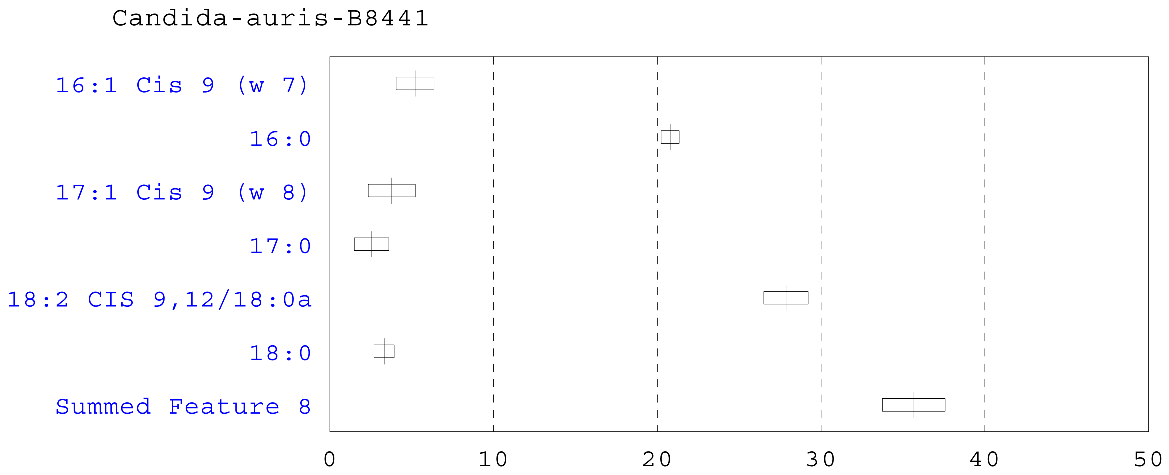

Figure S7. Cellular FA compositions of *C. auris* strain B8441 (Mean (%) ± SD)

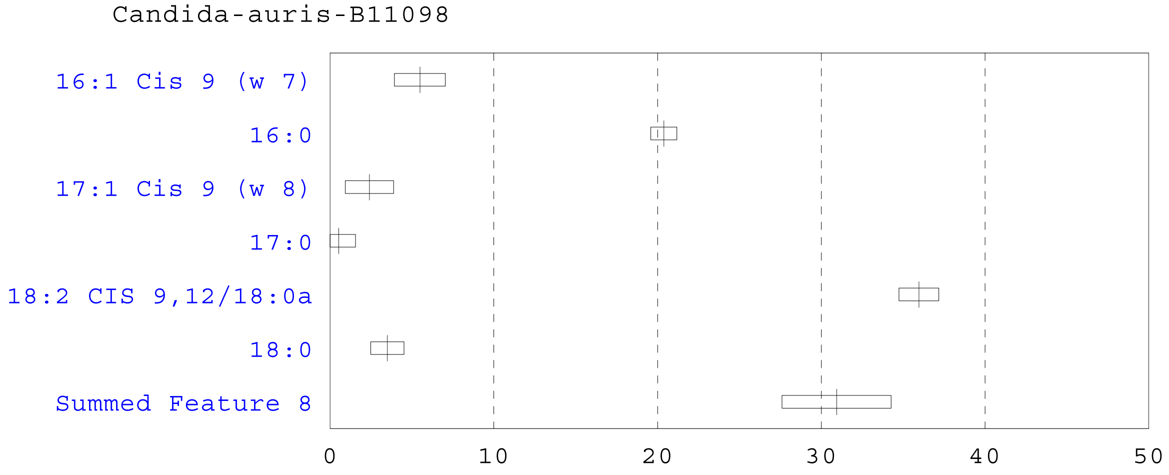

Figure S8. Cellular FA compositions of *C. auris* strain B11098 (Mean (%) ± SD)

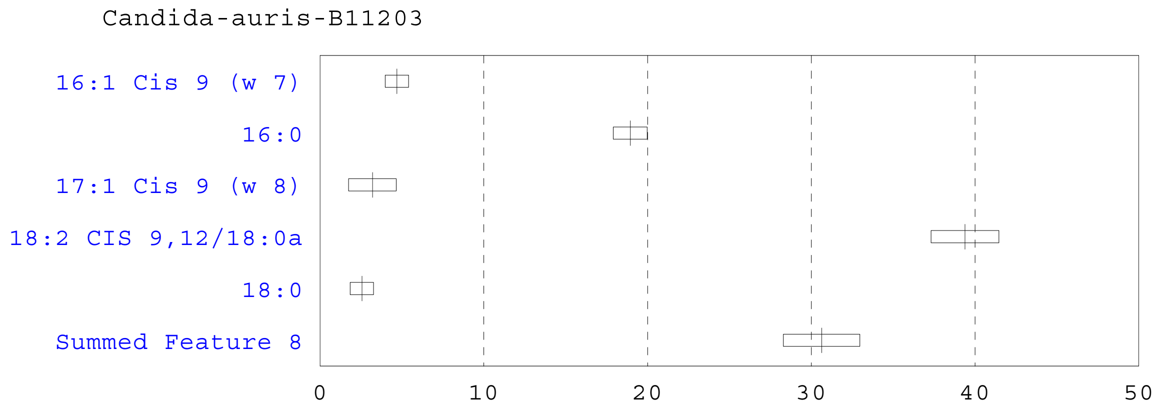

Figure S9. Cellular FA compositions of *C. auris* strain B11203 (Mean (%) ± SD)

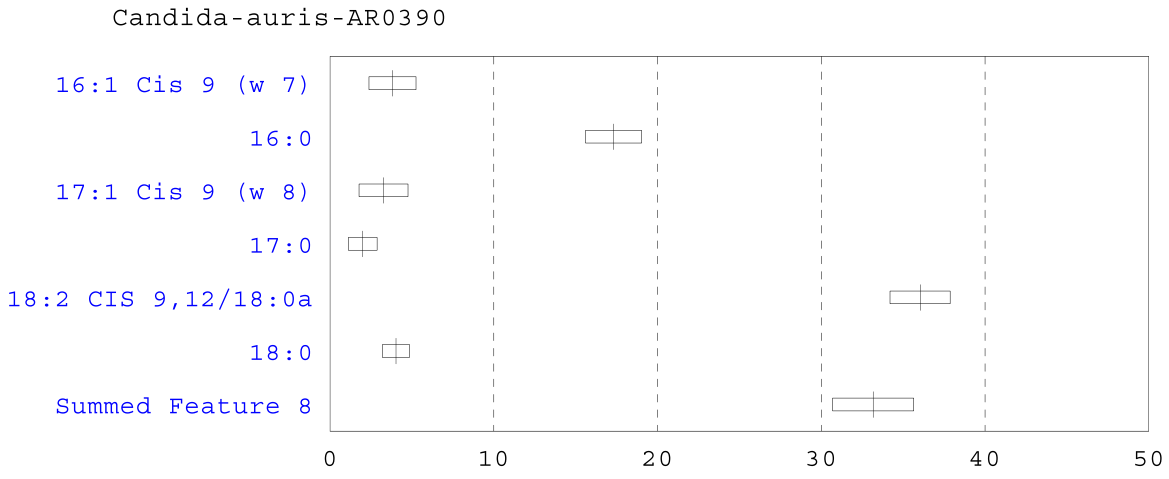

Figure S10. Cellular FA compositions of *C. auris* strain AR0390 (Mean (%) ± SD)

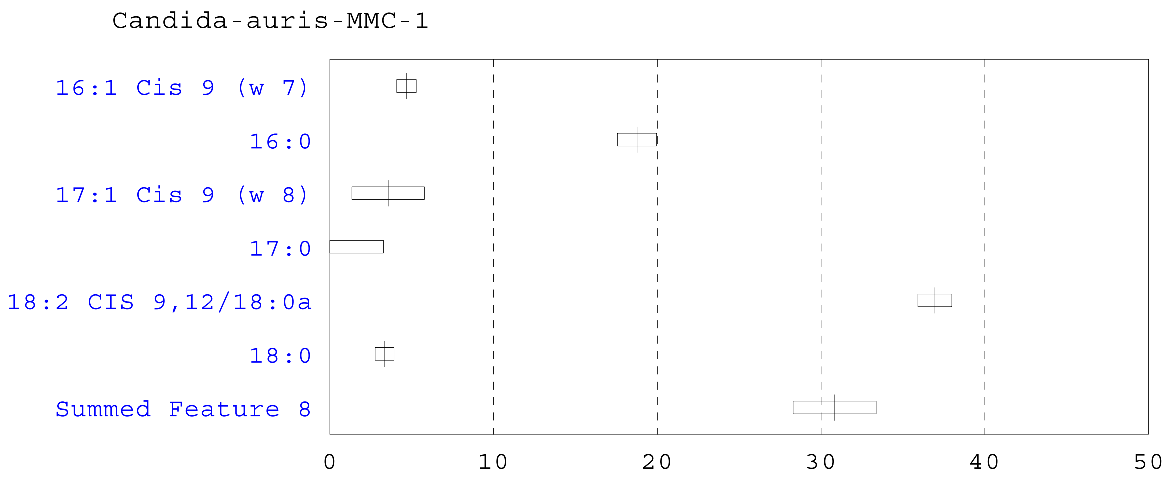

Figure S11. Cellular FA compositions of *C. auris* strain MMC-1 (Mean (%) ± SD)

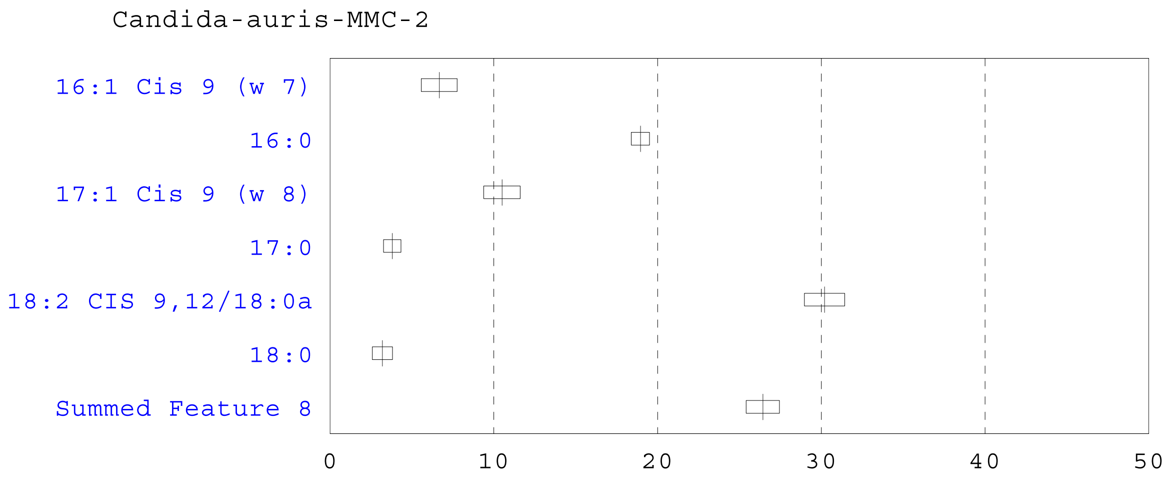

Figure S12. Cellular FA compositions of *C. auris* strain MMC-2 (Mean (%) ± SD)

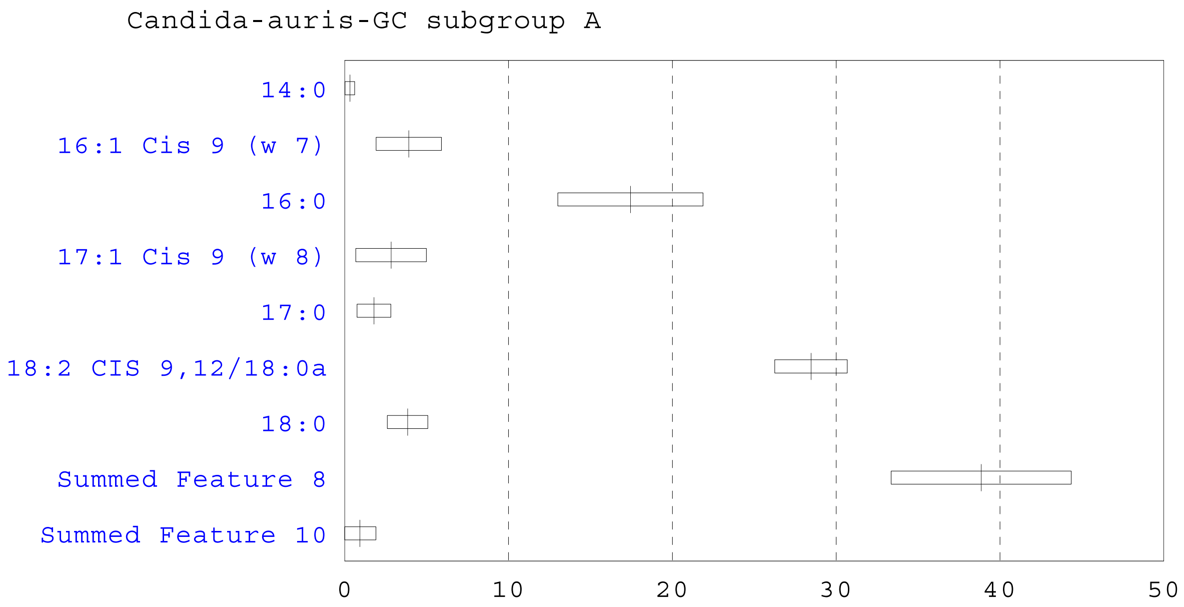

Figure S13. Cellular FA compositions of *C. auris* GC subgroup A (Mean (%) ± SD)

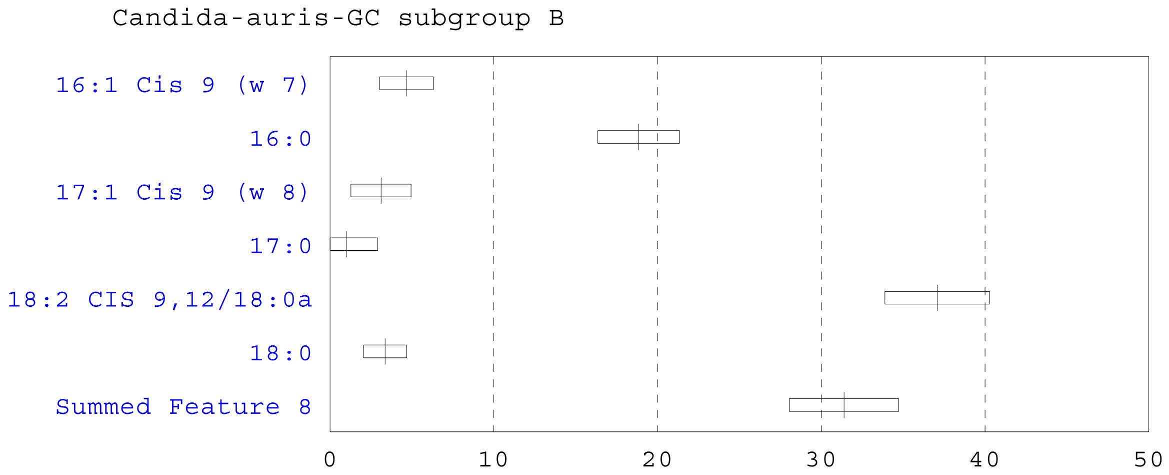

Figure S14. Cellular FA compositions of *C. auris* GC subgroup B (Mean (%) ± SD)

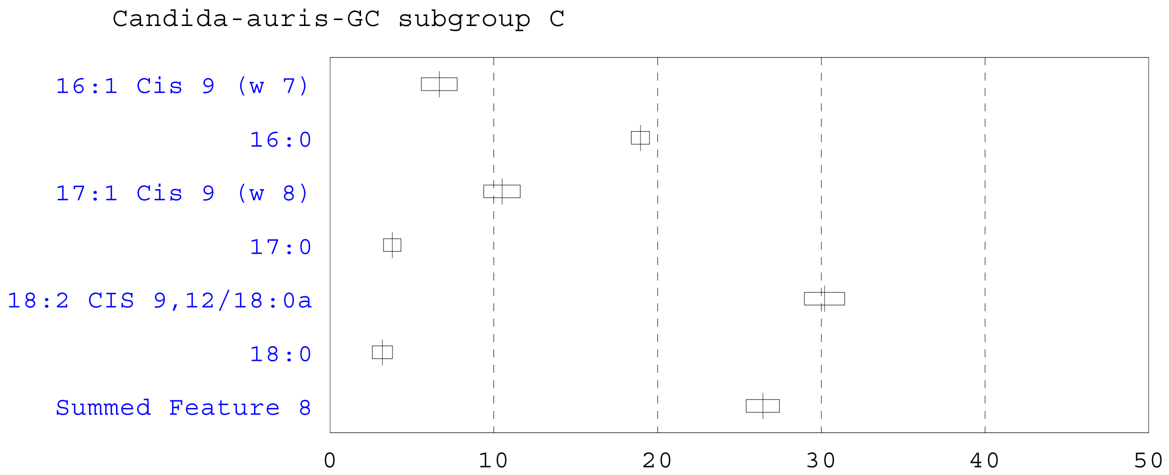

Figure S15. Cellular FA compositions of *C. auris* GC subgroup C (Mean (%) ± SD)

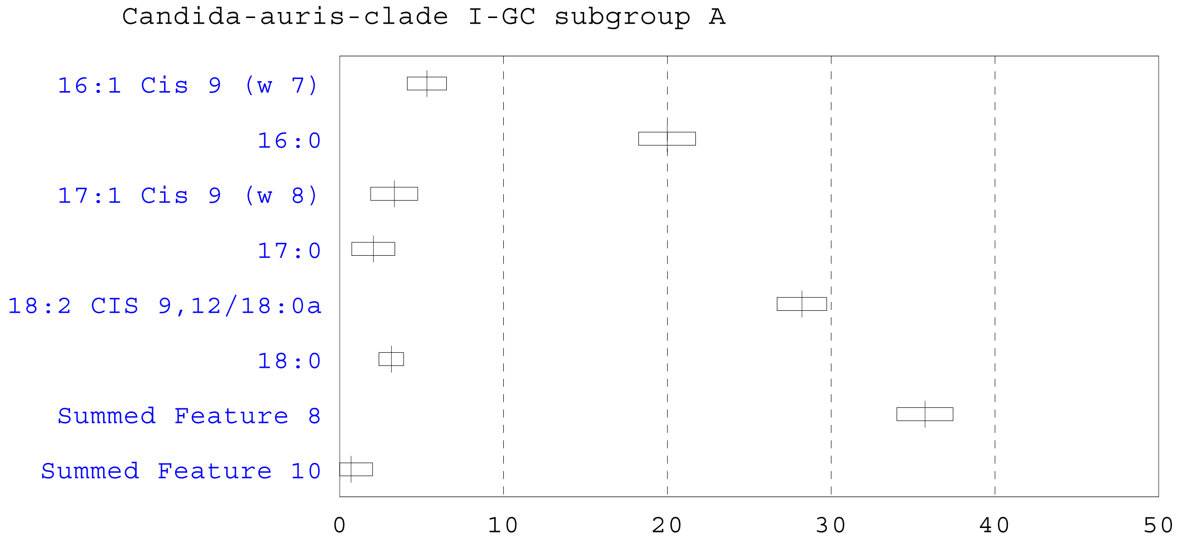

Figure S16. Cellular FA compositions of *C. auris* clade I – subgroup A

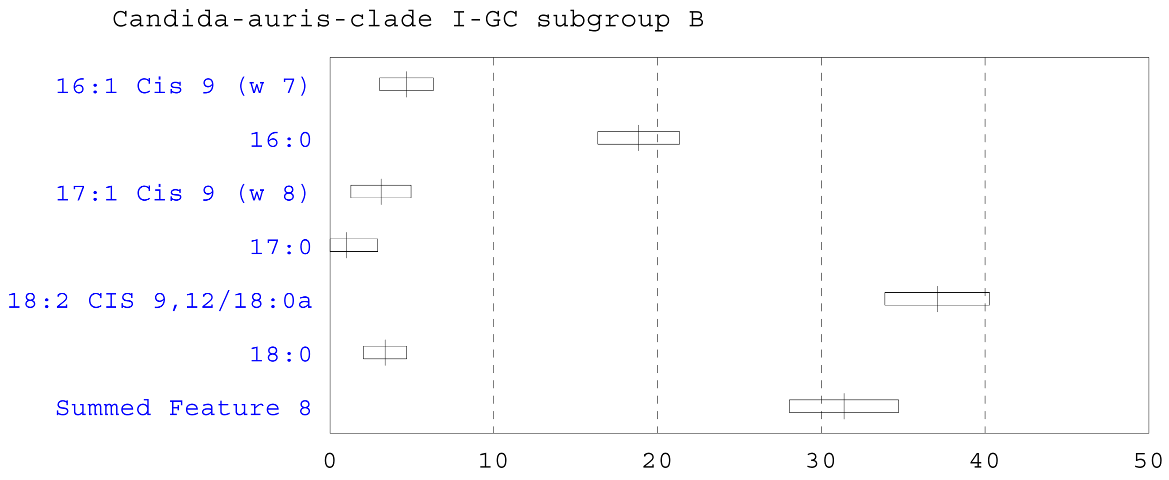

Figure S17. Cellular FA compositions of *C. auris* clade I – subgroup B

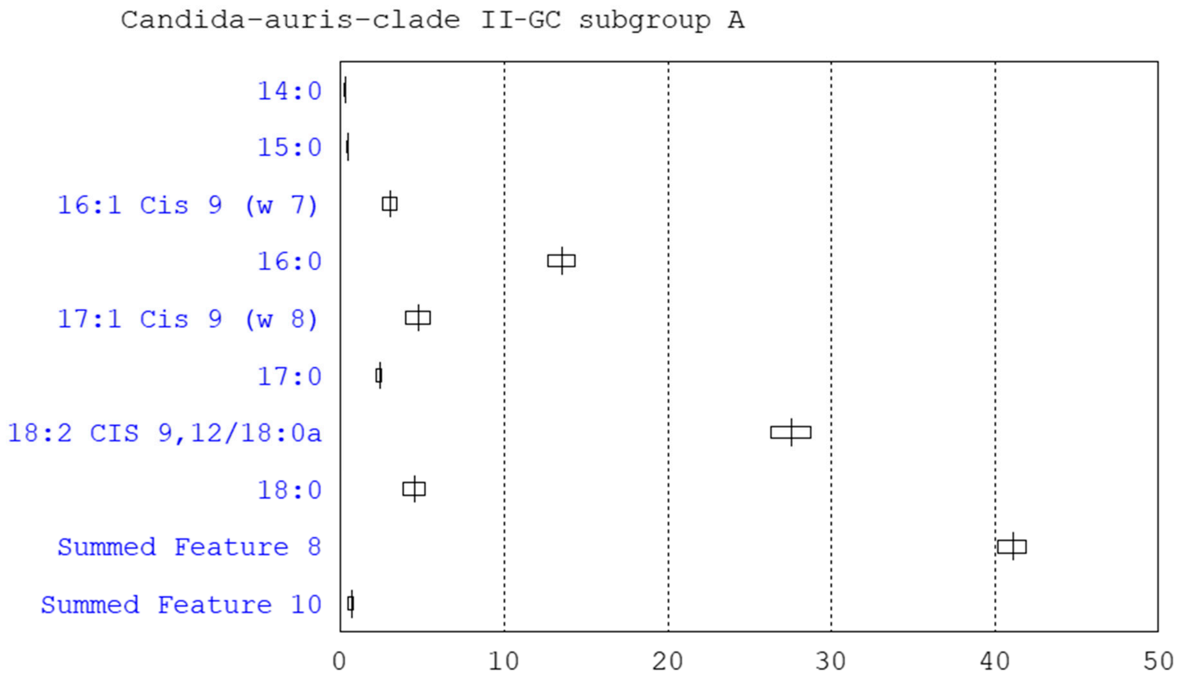

Figure S18. Cellular FA compositions of *C. auris* clade II – subgroup A

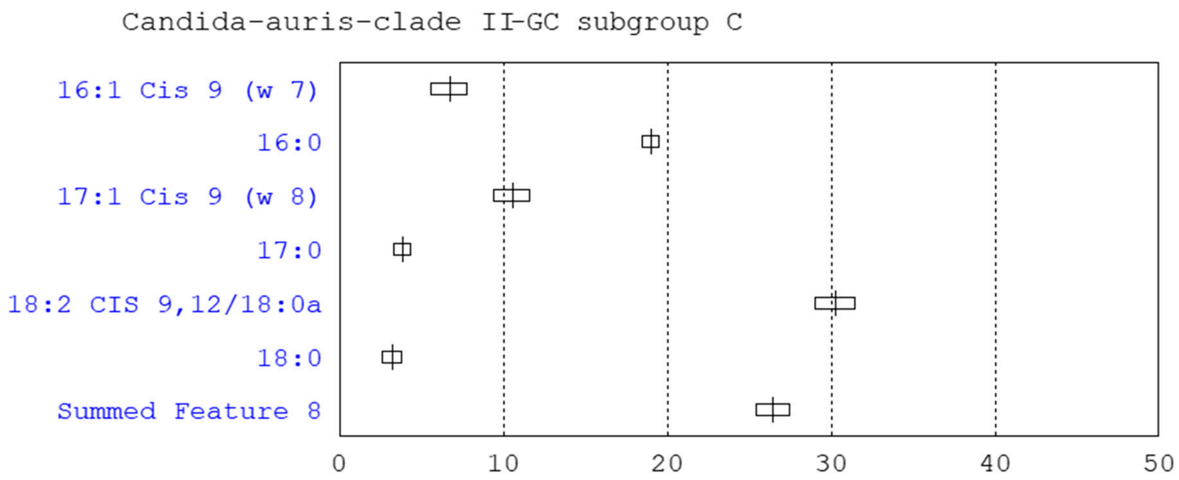

Figure S19. Cellular FA compositions of *C. auris* clade II – subgroup C

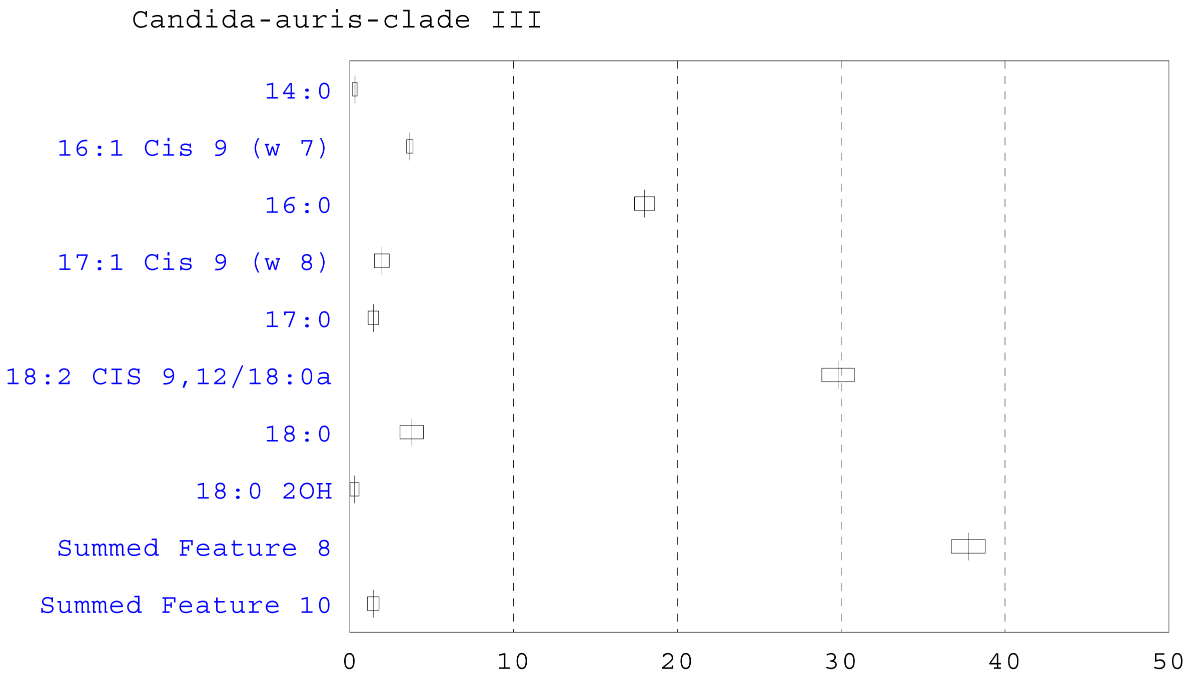

Figure S20. Cellular FA compositions of *C. auris* clade III

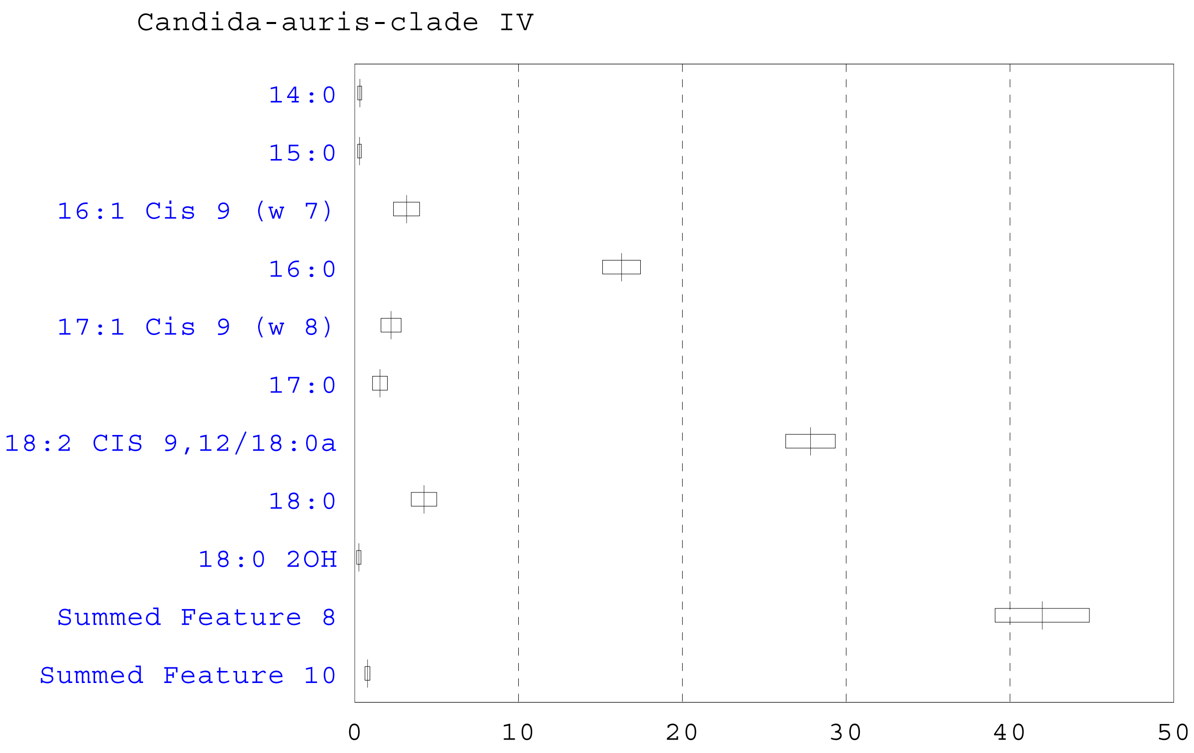

Figure S21. Cellular FA compositions of *C. auris* clade IV
